# Authors’ Response

**DOI:** 10.4269/ajtmh.19-0535b

**Published:** 2019-11

**Authors:** Hardeep Singh Malhotra, Ravindra Kumar Garg, Ravi Shekhar Jaipuriar, Imran Rizvi, Neeraj Kumar

**Affiliations:** Department of Neurology; King George Medical University; Lucknow, India; E-mails: drhsmalhotra@gmail.com, garg50@yahoo.com, jaipuriarshekhar@gmail.com, imranrizvi09@gmail.com, drneeraj2903@gmail.com

Dear Sir,

We thank the author for his comments on the immune status in patients with tuberculous meningitis.^1^ We agree that being tested negative for HIV infection does not mean that there is a perfect state of immunocompetence.^2^ We believe that immune dysregulation in any form, primary or secondary, can alter the course of tuberculous meningitis and affect its outcome. In view of the paucity of literature in this area, we are currently studying the prevalence of primary immunodeficiencies in patients with tuberculous meningitis, after having ruled out the commoner secondary immunodeficiencies in addition to HIV infection, including hepatitis C virus and hepatitis B virus infections, diabetes mellitus, and hepatic dysfunction.^3,4^ We are evaluating humoral (antibody and complement aberrancies) as well as cellular (T-cell and B-cell disorders) immune dysfunction. We expect that these studies will add to our understanding of immunodeficiency and tuberculous meningitis.
